# Assessment of the Impact of the Rotavirus Vaccine Against Severe Rotaviral Diarrhea in Uzbekistan

**DOI:** 10.1007/s44197-023-00126-z

**Published:** 2023-06-10

**Authors:** Erkin Musabaev, Umed Ismailov, Nargiz Ibadullaeva, Aziza Khikmatullaeva, Ilham Norbaev, Umar Primov, Dilorom Ahrarova, Saidkhon Sharapov, Ulugbek Yusupov, Renat Latipov

**Affiliations:** 1 Research Institute of Virology, Tashkent, Uzbekistan; 2 Fund of the State Medical Insurance of Jizzakh Region, Jizzakh, Uzbekistan; 3 Bukhara Regional Infectious Diseases Hospital, Bukhara, Uzbekistan; 4 4th City Pediatric Infectious Diseases Hospital, Tashkent, Uzbekistan; 5 Bukhara State Medical Institute, Bukhara, Uzbekistan

**Keywords:** Rotavirus, Vaccination, AGE, RVGE, UMV, Tashkent, Bukhara

## Abstract

**Background:**

This article presents the results of a long-term study of the impact of rotavirus vaccination in Uzbekistan. Uzbekistan is the first country in the Central Asian region to introduce rotavirus vaccination into the national compulsory vaccination calendar. The study aimed to evaluate the impact of rotavirus vaccination on hospitalizations due to all-cause AGE and RVGE in children < 5 years of age in Uzbekistan.

**Methods:**

Detection of rotavirus antigen was performed using Rotavirus-Antigen-IFA-BEST "Vector Best" kit (Novosibirsk, Russia).

**Results:**

The total of 20,128 children under 5 years of age were hospitalized in sentinel hospitals with a diagnosis of acute gastroenteritis during the study period (2019–2020). Of this number of children, 4481 children (22.2%) were included in the study. Of 4481 children, 367 (8.2%) children tested positive for rotavirus. In our study, decrease in the rotavirus rate was noted in all age groups. The peak of rotavirus positivity occurred in the months of January and February.

**Conclusion:**

The average rotavirus-positive rate in the period (2019–2020) was 8.2% and the absolute percentage decrease was 18.1% compared to the pre-vaccination period (2005–2009) where the rotavirus-positive rate was 26.3%. The percentage of prevented cases averaged 68.8%.

## Introduction

Childhood diarrheal diseases remain a major public health problem due to high morbidity, development of severe forms, complications, and high mortality. Rotaviruses are the most common cause of severe diarrhea in children under 5 years of age and globally 215,000 deaths related to this infection occurred in 2013 [1]. In Uzbekistan, tens of thousands of children are hospitalized each year for rotavirus infection, which accounts for the social importance of the problem. According to studies conducted by the Research Institute of Virology in Uzbekistan [2], rotavirus infection is observed as a cause in 26–41% of cases of acute diarrhea, causing more than 5000–10,000 hospitalizations in children under 5 years of age each year. The annual increase in illness and the resulting increase in hospitalizations cause a significant public outcry and an enormous economic burden. A 2007 study found that the cost per child hospitalized for diarrhea in Uzbekistan was US$77.8 (a current figure of US$96.3 when adjusted for inflation in 2010), with the Government covering 89.0 percent of the cost [3]. Based on the above results, it is concluded that rotavirus infection is widespread in Uzbekistan and causes a large number of severe cases of gastroenteritis in children under 5 years of age; the economic burden of hospitalizing children reaches half a million US dollars. As a result, vaccination against rotavirus infection of children aged 2 and 3 months started in June 2014. By the support of GAVI, Uzbekistan introduced the Rotarix vaccine (GlaxoSmithKline Biologicals, Rixensart, Belgium) into the childhood immunization program. The results of monitoring vaccination introduction show that the coverage of the rotavirus vaccine was about 99.8% in 2015–2018. The active hospital sentinel surveillance for both acute gastroenteritis (AGE) and rotavirus gastroenteritis (RVGE) was conducted in Uzbekistan from 2005 to 2009, and resumed in January 2014 after the introduction of RVI vaccination.

The study aimed to evaluate the impact of rotavirus vaccination on hospitalizations due to all-cause AGE and RVGE in children < 5 years of age in Uzbekistan.

## Materials and Methods

### Study Design

An active hospital sentinel surveillance based on generic protocol for rotavirus hospital-based surveillance [4] was established to determine the impact of the vaccination, on hospitalization due to rotavirus diarrhea (RVGE) and all-cause diarrhea (AGE). The impact was evaluated by comparing the AGE and RVGE numbers and rates registered in surveillance before (2005–2009) and after vaccine introduction into UMV (2019–2020). These data were interpreted in conjunction with the vaccine coverage estimates (routinely collected by the Ministry of Health) for all areas served by hospitals. Three periods were considered for the study: the period before vaccination in 2005–2009, the period of introduction of vaccination (transition period) in 2014–Dec 2015, and the period of long-term effect (2019–2021).

### Description of Surveillance and a Source of the Cases

The active hospital-based surveillance for acute gastroenteritis (AGE, ICD-10 A.09) cases among children under the age of 5 years admitted to the hospital was established in 2005. Among the admitted patients with AGE, a stool sample was collected from all/systematically (every 3rd AGE A.09) sampled subjects for rotavirus detection (RVGE, ICD-10 A08.0). The same approach was used for surveillance in the hospitals during post-introduction of RV vaccine. A local coordinator in the hospital or a member of the study group in each hospital reviewed the logs of admitted patients to identify children with gastroenteritis admitted to the hospital who met the criteria for inclusion in the surveillance program in both the pre-vaccination and post-vaccination periods. Information was collected as stated in the case report form developed for the study.

### Informed Consent

As a part of the surveillance, informed consent was obtained from a subset of children admitted with AGE for testing of RV in the stool. Since all eligible children were younger than 5 years, informed consent was received from parents (father or mother) or official caregivers. Filled forms of informed consent were kept with a medical record (disease history) of each child.

### Laboratory Tests

From each child enrolled in a surveillance program and from whom informed consent was taken and stool samples were collected, the sample was divided into three aliquots at the Research Institute of Virology in Tashkent. One aliquot was analyzed for the presence of rotavirus antigen in the Research Institute of Virology, using ELISA test-kit Rotavirus-Antigen-EIA-BEST «Vector Best» (“Novosibirsk, Russia”) and in compliance with the manufacturer's instructions. This kit shows 100% sensitivity, 93.2% specificity, and 98.8% reproducibility compared to reference kits (IDEA™ Rotavirus (DAKOCytomation, UK) [5]. Evaluation of the results was conducted following with the manufacturer instructions for use attached to the kit. The other two aliquots of each sample were frozen and kept until analysis was completed (as a backup). The laboratory procedure was the same both in the pre- and post-vaccination periods.

### Inclusion and Exclusion Criteria

The following inclusion and exclusion criteria were used during the study. The same criteria were followed throughout the period.

**Children were included in the study if they meet all of the following criteria**:

- The child was resident of the Republic of Uzbekistan
- Age < 5 years at the time of admission
- The child was born in May 2014 or later
- A child with acute diarrhea gastroenteritis (AGE, A.09), which was defined as (the same definition used in the previous surveillance). Passage of three or more abnormal loose stools in any period of 24 h and for which the child was admitted to the hospital.
- A child was able to provide stool sample during the first 48 h of hospitalization and tested for rotavirus

**Children were not included in the study if any of the following conditions were present**:

- The child did not live in the Republic of Uzbekistan
- The child was born before May 2014
- The child was transferred from another hospital after staying there for > 48 h
- The child had diarrhea for > 7 days prior to the visit to the hospital
- The child was included in surveillance earlier in connection with the same case of gastroenteritis
- The child was not able to produce stool during the hospitalization period

Similar inclusion and exclusion criteria were applied for all the subjects enrolled in the surveillance and for both the pre- and post-vaccination period. About 8000 diarrhea cases in Tashkent and 1500 cases in Bukhara among children under 5 years old are hospitalized annually. To reduce human and material costs, it was included every fifth case in Tashkent and every third case in Bukhara. The order of inclusion was determined based on records in the registration log at the admission ward. All eligible patients were included in the surveillance.

### Sample Size and Statistical Power

For impact assessment, there was no pre-determined sample size. Instead, the prospective active surveillance of hospital admissions of children aged < 5 years due to rotavirus gastroenteritis was continued for at least 3 calendar years (as per WHO guidelines) after the introduction of the vaccine to have sufficient information to compare the results before the introduction of the vaccine with the results after its implementation. The rotavirus positivity rate was presented by mean (M) in sentinel sites, age groups, and comparative groups. The variance of a mean was presented by the standard error (m, the standard deviation, and dividing it by the square root of the sample size). The statistical significance (p) of the changes in proportion of the rotavirus positivity before and after vaccine introduction, as well as between groups was determined by Fisher’s exact test. Both the proportion and their 95% CI will be calculated.

### Data Collection and Variables

The following data were collected:

**Patient information**: Name of the hospital submitting information, information receipt date, identification number, age (months), and gender, for children born after May 1, 2014, name of each health center or a private clinic, where the child has received vaccinations.

**Clinical data:** Body temperature, vomiting (yes/no), the number of vomiting/seizures during the 24 h, duration of vomiting (days), diarrhea (yes/no), the number of diarrhea episodes/24 h, duration of diarrhea (days), episode resolution (discharge or death), duration of hospital stay (days), intravenous infusion, the degree of dehydration assessed by a physician at admission to the hospital.

**Laboratory data:** Date of stool samples collection, date of stool sample delivery to the laboratory, date of sample testing for rotavirus, any bacteria found in the stool?, any parasites found in stool?, date of rotavirus antigen detection in the stool using ELISA.

Data for all children included in the surveillance program were collected and entered using paper case report forms by a trained field surveillance coordinator. Efforts were made to ensure getting the most complete and accurate data. Data were entered in the Excel database. Statistical program («SPSS 21») was used for data analysis. Paper case report forms were filled in by surveillance coordinators. These forms will further be transcribed into the electronic database, password protected. Quality control of information in the database was carried out every 15 days. The Research Institute of Virology’s staff was responsible for the management, interpretation, analysis, and dissemination of data.

### Data Analysis Plan

The vaccination impact was evaluated based on analysis of the total number of hospitalizations due to all-cause diarrhea (AGE A.09) during two periods: baseline (2005–2009, i.e., 5 years of pre-vaccination data) and post-vaccination (2019–2020, i.e., 2 years of post-vaccination data) periods. Since vaccination started in June 2014 in UMV, we will use the time until December 2015 as a transition/buffer period for the vaccine to take effect and increase in coverage rate across the country. The pre-vaccination period included published data from surveillance carried out during 2005–2009 [1]. This surveillance has been implemented based on the same study protocol and in the same sentinel sites. Our post-vaccination study for RVGE began from January 2019 to February 2021. Surveillance sites will keep the same operational procedures to minimize possible bias linked with the immunization program start. The statistical significance (p) of the changes in the proportion of rotavirus positivity before and after vaccine introduction, as well as between groups was determined by Fisher’s exact test. Both the proportion and their 95% CI will be calculated.

The total number of hospitalization with AGE among children under 5 years of age and in different age strata was the denominator for any proportion calculation, while the number of RVGE was the numerator. The RVGE positivity (%) was calculated by the following formula: positive RVGE cases/all patients who have been tested for RVGE. The number of hospitalizations was compared for specific age groups (e.g., 0–5 months, 6–11 months, 1 year, 2 years, 3 + 4 years, all children < 5 years) and seasons (based on published data collected before the introduction of the vaccine, about months in which a large number of recorded cases of rotavirus infection detected, compared to months with low positive case prevalence of rotavirus infection). To obtain such information, hospital admission logs were reviewed. Adjustments were made for the percentage of children not covered by the surveillance program.

## Ethical Considerations

The study protocol was submitted to the Ethics Committee of the Ministry of Health of the Republic of Uzbekistan for approval. The parents or legal guardians of participating children provided written consent after being informed about the study goals and objectives and the conditions for and potential benefits of their children’s participation. Vulnerable children (such, as orphans living in orphanages and boarding schools) were excluded. All research data were coded. Access to data was limited to the principal researchers and study team only.

## Results

The total number of 20,128 children under 5 years of age were hospitalized in sentinel hospitals with a diagnosis of acute gastroenteritis (AGE ICD-10 A.09) during the study period. Of this number of children, 4481 children (22.2%) who met the study criteria were included in the study. Of them, 3268 (72.9%) were included in Tashkent and 1213 (27.1%) were included in Bukhara (Table 1).

**Table 1:** Total number of hospitalized patients with AGE, of which those tested for RVGE (0–5 years old)

Region	Total hospitalized with AGE	Tested for RVGE	Positive of RVGE	%
Tashkent	16,459	3268	276	8.4
Bukhara	3669	1213	90	7.5
Total	20,128	4481	367	8.2

Of 4481 children, 367 (8.2 ± 0.4%) children tested positive for rotavirus (Table 2). Certain fluctuations were observed in the proportion of rotavirus positivity in tested children: from 1.5 ± 1.1% to 17.2 ± 2.8%. Since all infectious diseases hospitals in the republic were re-purposed into centers for fighting COVID-19 infection, material sampling was not possible in February 2020 in Tashkent and in the period from April to November 2020, and February 2021 in Bukhara. There is a surveillance data gap from April to November 2020 (due to the COVID-19 pandemic).

**Table 2:** The number of hospitalized children with acute gastroenteritis (AGE, A09) included in the study by years and study sites, and the average distribution of rotavirus (RVGE, A.08.0) cases among children under 5 years of age

Year	Month	Fourth city infectious diseases hospital of Tashkent			Bukhara district pediatric infectious diseases hospital			Total AGE hospitalizations		
		Total AGE	RV positive	%	Total AGE	RV positive	%	Total AGE	RV positive	M ± m
2019	January	118	21	17.8	68	11	16.2	186	32	17.2 ± 2.8
	February	78	15	19.2	55	7	12.7	133	22	16.5 ± 3.2
	March	85	14	16.5	62	7	11.3	147	21	14.3 ± 2.9
	April	161	7	4.3	81	2	2.5	242	9	3.7 ± 1.2
	May	158	3	1.9	84	1	1.2	242	4	1.7 ± 0.8
	June	195	6	3.1	116	1	0.9	311	7	2.3 ± 0.8
	July	182	4	2.2	118	1	0.8	300	5	1.7 ± 0.7
	August	165	5	3.0	90		0.0	255	5	2.0 ± 0.9
	September	132	6	4.5	79	3	3.8	211	9	4.3 ± 1.4
	October	146	14	9.6	92	8	8.7	238	22	9.2 ± 1.9
	November	127	14	11.0	91	10	11.0	218	24	11.0 ± 2.1
	December	108	17	15.7	62	8	12.9	170	25	14.7 ± 2.7
2020	January	83	15	18.1	73	12	16.4	156	27	17.3 ± 3.0
	February				59	9	15.3	59	9	15.3 ± 4.7
	March	29	4	13.8	31	4	12.9	60	8	13.3 ± 4.4
	April	46	3	6.5				46	3	6.5 ± 3.7
	May	91	2	2.2				91	2	2.2 ± 1.5
	June	122	3	2.5				122	3	2.5 ± 1.4
	July	130	2	1.5				130	2	1.5 ± 1.1
	August	137	3	2.2				137	3	2.2 ± 1.3
	September	186	11	5.9				186	11	5.9 ± 1.7
	October	192	23	12.0				192	23	12.0 ± 2.3
	November	163	20	12.3				163	20	12.3 ± 2.6
	December	146	20	13.7	32	4	12.5	178	24	13.5 ± 2.6
2021	January	155	25	16.1	20	3	15.0	175	28	16.0 ± 2.8
	February	133	19	14.3				133	19	14.3 ± 3.0
	Total	3268	276	8.4	1213	91	7.5	4481	367	8.2 ± 0.4

**Fig. 1:**
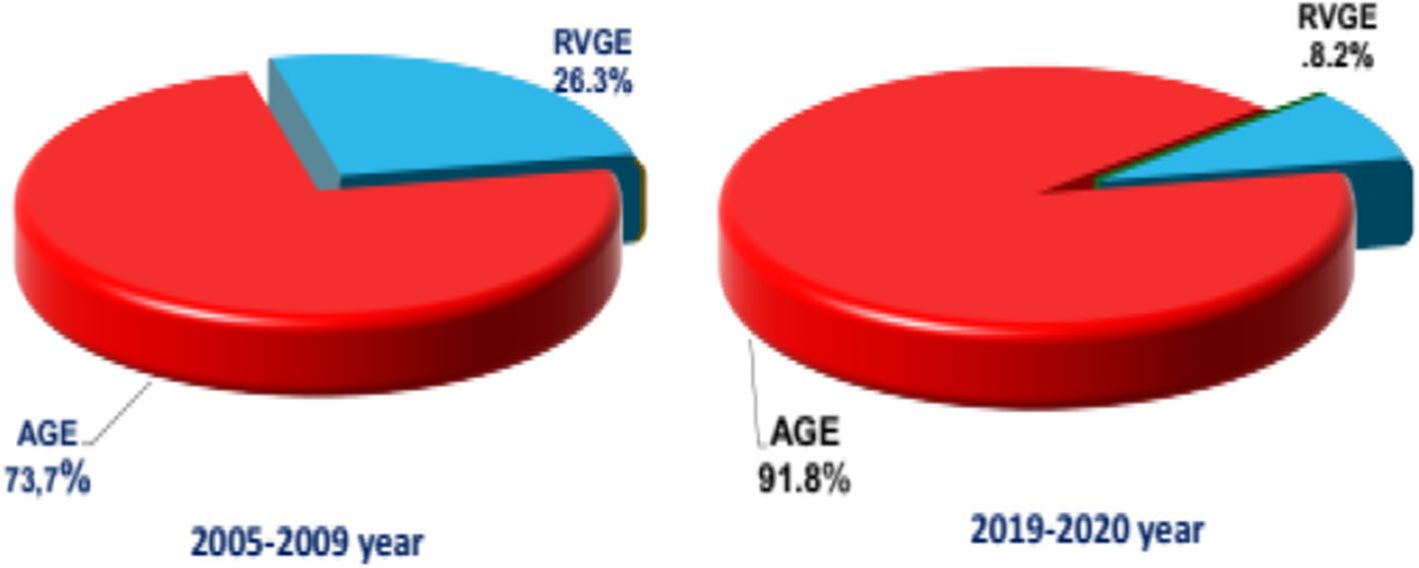
The incidence of RVGE (A.08.0) among hospitalized with AGE (A.09) in children under 5 years of age

For non-availability of RV testing (during 2010–2018), two full calendar years post-vaccination (2019–2020) data were compared with that of 5 years of historical data (2005–2009) for RVGE in the under-5-years age group (Fig. 1).

Of 4481 children, 2471 were boys and 2010 were girls (Table 3). The boys-to-girls ratio was 1.2 to 1. Average rotavirus positivity was 8.3 ± 0.55% in boys and 8.06 ± 0.61% in girls. There was no statistically significant difference between boys and girls.

**Table 3:** Positive cases of rotavirus by gender in children included in the study sites, and the average distribution of rotavirus infection (RVGE, A.08.0) in acute gastroenteritis (AGE, A09) cases in hospitalized children under 5 years of age

Gender	Sentinel site	Number of AGE hospitalizations (RV positive)	RV positive%,	Standard error of the mean	Significance of differences, *p*
Female	Tashkent	1503 (123)	8.10	0.71	0.74
	Bukhara	507 (39)	7.70	1.18	
	Total	2010 (162)	8.06	0.61	
Male	Tashkent	1765 (153)	8.67	0.67	
	Bukhara	706 (52)	7.37	0.98	
	Total	2471 (205)	8.30	0.55	
Total	Tashkent	3268 (276)	8.45	0.49	
	Bukhara	1213 (91)	7.50	0.76	
	Total	4481 (367)	8.19	0.41	

The average age of children was 17.12 ± 0.65 months, 17.86 ± 0.726 months in Tashkent, and 14.87 ± 1.407 months in Bukhara (Table 4). The difference between cities was not statistically significant.

**Table 4:** Age of rotavirus-positive children included in the study, by years and study sites, and average proportion of rotavirus infection (RVGE, A.08.0) cases in children under 5 years of age

Gender	Sentinel site	Number RV positive	Age, months, average value	Standard error of mean	Significance of differences, *p*
F	Tashkent	123	17.49	1.103	0.426
	Bukhara	39	13.54	2.102	
	Total	162	16.54	0.984	
M	Tashkent	153	18.16	0.965	
	Bukhara	52	15.87	1.898	
	Total	205	17.58	0.867	
Total	Tashkent	276	17.86	0.726	
	Bukhara	91	14.87	1.407	
	Total	367	17.12	0.65	

During the entire observation period, there were no fluctuations in the average age value, both by years and by sites. During all observation periods, the average age of children in Bukhara district hospital was a little lower; it means that being a regional center, this hospital was receiving children in more serious conditions referred from other district hospitals. This may have an impact on the increase in the percentage of rotavirus patients in Bukhara district hospital.

Having studied the distribution of the frequency of rotavirus detection by age groups, it was found that the rates of rotavirus detection between age groups do not differ, not statistically significant (*p* = 0.585) (Table 5).

**Table 5:** Distribution of rotavirus-positive cases by age groups

Age groups	Number total AGE	Mean of RVGE %	Standard error of the mean	Significance of differences, *p*
0–5 months	620	7.58	1.06	
6–11 months	1205	8.22	0.79	
12 –23 months	1408	8.95	0.76	0.585
24–60 months	1248	7.61	0.75	
Total	4481	8.19	0.41	

The analysis of annual distribution shows pronounced seasonal fluctuations. The peak of rotavirus positivity occurs in the months of January and February. Minimum values may be observed in the summer months.

**Fig. 2:**
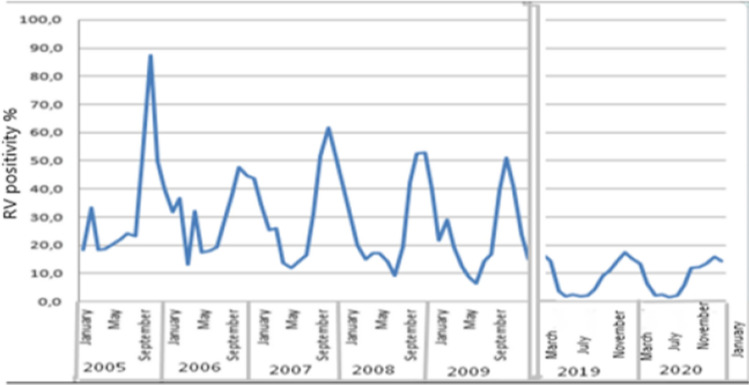
Seasonal distribution of cases of rotavirus gastroenteritis before and after vaccination (Note: time gaps from 2010 to 2013 and 2018 because not tested for RVGE)

Changes in the occurrence of rotavirus are also noted when examining the age composition. Thus, during the period of vaccine introduction, there was a decrease in the rotavirus rate among children under 2 years of age, which is a logical consequence of vaccinating children at an early age, whereas in our study, the same level was noted in all age groups. The seasonal distribution of rotavirus gastroenteritis cases also shows changes. The pronounced seasonal peaks in the pre-vaccination period decreased significantly immediately after the introduction of the vaccine and became less pronounced in the period under study. In addition, the peak of morbidity shifted from the month of October to the January–February months, as in many countries in the European region and America (Fig. 2).

## Discussion

The results of our study, in comparison with historical data, show certain changes in the epidemiology of rotavirus gastroenteritis in children under 5 years of age after the introduction of rotavirus vaccination into the national vaccination schedule.

The results of previous studies based on a similar study protocol were used for comparison. Thus, the mean rate of positive cases for rotavirus in the pre-vaccination period (2005–2009) was 26.3 ± 0.4% with little difference between cities [2]. In our study, this rate decreased to 8.2 ± 0.4%. The absolute percentage decrease was 18.1%. The percentage of prevented cases averaged 68.8% (2005–2009) among all the AGE, and the frequency of RVGE was 26.3% (100%); in 2019–2021 among all the AGE, the frequency of RVGE was 8.2% (31.2%); 100%–31.2% = 68.8%.

In addition, it should be noted that the Rotarix vaccine, GSK, was the first to be introduced into the National Vaccination Calendar. The vaccine was introduced based on funding from GAVI. Subsequently, since January 2020, the government decided in favor of the BRV-PV (Rotasil) Serum Institute of India vaccine, a decision made out of financial priorities.

This article presents the results of a long-term study of the impact of rotavirus vaccination in Uzbekistan. Uzbekistan is the first country in the Central Asian region to introduce rotavirus vaccination into the national compulsory vaccination calendar. The rotavirus vaccine is fundamentally different from vaccines against other infections. The vaccine does not protect against infection in the way we are used to seeing vaccines. The vaccine protects against the severity of the disease, not the disease itself. A vaccinated child can and most likely will be re-infected with rotavirus, but the reinfection will be milder and not require hospitalization [6–8]. At the same time, rotavirus infection is responsible for only a quarter of all gastroenteritis cases in children, so accurate data on the proportion of rotavirus infection in the pre-vaccination and post-vaccination rates of acute diarrhea are necessary to arbitrate the effectiveness of vaccination.

The COVID-19 pandemic has impacted our study, which should be considered when analyzing the results. Thus, during the pandemic in Uzbekistan, lockdowns were introduced several times. Lockdown is an artificial intervention that affects the natural epidemiological course of the disease, and naturally, when analyzing the effect of vaccination, this must be considered. Therefore, periods of lockdowns introduced due to COVID-19 were excluded in our study. This resulted in gaps in data collection and the post-vaccination period only included complete data for 1 year (2019), half of 2020, and some months from 2021, this makes it possible to compare the natural epidemiological manifestation without the influence of pandemic measures.

Impact assessment can be affected by changes associated with population or hospitals in the period after the introduction of the vaccine compared with the period prior to its implementation, regardless of the rotavirus vaccine. These changes may include a change in the area served by a particular hospital for children with acute diarrhea; changes in the number of cases of outpatient treatment of acute diarrhea in children, which entails a reduction in the number of children entering treatment; changes in the number of beds available in the hospital for children with acute diarrhea; changes in admission procedures to the hospital (for example, the requirement for presentation of the direction of the clinic); changes in the cost of treatment bared by parents; as well as other factors, such as the common behaviors associated with active treatment for medical care among parents of children with acute diarrhea. The performance of the vaccine and the reduction of disease (both AGE and RVGE and their changes in different age strata) were seen in both numbers and rates of hospitalizations due to diarrhea with a positive test for rotavirus with that of hospitalizations for diarrhea with a negative test result rotavirus. Hence, the impact of the vaccination was assessed both for AGE and RVGE.

Data show that the current vaccination rate in Uzbekistan is 98.8% in 2015–2018 years [9]. The percentage of prevented cases averaged 68.8%. This figure is not far from the best European and U.S. rates of 75–94% [10–13] and much better than African rates of 39.3–48.3% [14–17]. However, the effectiveness of vaccination will continue to increase because the study did not consider herd immunity, which according to recent data, plays quite a significant role in protecting against rotavirus infection [18, 19].

## Conclusion

The mean rate of positive cases for rotavirus in the period (2019–2020) was 8.2% and the absolute percentage decrease was 18.1% compared to the pre-vaccination period (2005–2009) where the rotavirus-positive rate was 26.3%. The percentage of prevented cases averaged 68.8%. The role of vaccination in decreasing the proportion of rotavirus is particularly clearly indicated by the shift in the age composition we observed toward the "maturation" of the peak of the infection and the disappearance of the role of age in the prevalence of rotavirus in the long term. The vaccination against rotavirus is bearing fruit and the result obtained in the first stages can be considered a good start but requires further and deeper study of the issue.

### Limitations

The following limitations of the study should be noted. Comparisons were made using data from several studies conducted at different times, which leads to data gaps. However, the use of a single study protocol, the same sentinel sites, allows direct comparison of data.

The COVID-19 pandemic had its impact on the natural course of the epidemiological process, so lockdown periods were excluded from the study.

This raises the question of whether the decrease in the proportion of rotavirus we observe is a natural process caused by factors other than vaccination. This is not to be discounted, but it must be considered that the data obtained by Rheingans et al. [20] indicate that the proportion of rotavirus also increases as a country's income rises. This was demonstrated with success in comparisons between Uzbekistan, Kazakhstan, and Kyrgyzstan in our previous studies. Based on this, and because since 2009, Uzbekistan, according to the World Bank classification, moved from low-income to lower middle-income countries, one would expect that the share of rotavirus should have increased.

## Data Availability

Available and kept confidential.

## Abbreviations

AGE: Acute gastroenteritis
RVGE: Rotavirus gastroenteritis
ELISA: Enzyme-linked immunosorbent assay
UMV: Universal mass vaccination
GAVI: Global alliance for vaccines and immunization
